# Mean arterial pressure and mortality in patients with distributive shock: a retrospective analysis of the MIMIC-III database

**DOI:** 10.1186/s13613-018-0448-9

**Published:** 2018-11-08

**Authors:** Jean-Louis Vincent, Nathan D. Nielsen, Nathan I. Shapiro, Margaret E. Gerbasi, Aaron Grossman, Robin Doroff, Feng Zeng, Paul J. Young, James A. Russell

**Affiliations:** 1 Department of Intensive Care, Erasme Hospital, Université libre de Bruxelles, Route de Lennik 808, 1070 Brussels, Belgium; 2 Division of Pulmonary Disease, Critical Care and Environmental Medicine, Tulane University School of Medicine, New Orleans, LA 70112 USA; 3 Department of Emergency Medicine, Beth Israel Deaconess Medical Center and Harvard Medical School, Boston, MA 02215 USA; 4 SAGE Therapeutics Inc, Cambridge, MA 02142 USA; 5 Policy Analysis Inc, (PAI), Brookline, MA 02445 USA; 6 La Jolla Pharmaceutical Company, San Diego, CA 92121 USA; 7 Medical Research Institute of New Zealand, Wellington, 6021 New Zealand; 8 Division of Critical Care Medicine, St. Paul’s Hospital, Vancouver, BC V6Z 1Y6 Canada

**Keywords:** Mean arterial pressure, Acute circulatory failure, Multiple organ failure, ICU mortality

## Abstract

**Background:**

Maintenance of mean arterial pressure (MAP) at levels sufficient to avoid tissue hypoperfusion is a key tenet in the management of distributive shock. We hypothesized that patients with distributive shock sometimes have a MAP below that typically recommended and that such hypotension is associated with increased mortality.

**Methods:**

In this retrospective analysis of the Medical Information Mart for Intensive Care (MIMIC-III) database from Beth Israel Deaconess Medical Center, Boston, USA, we included all intensive care unit (ICU) admissions between 2001 and 2012 with distributive shock, defined as continuous vasopressor support for ≥ 6 h and no evidence of low cardiac output shock. Hypotension was evaluated using five MAP thresholds: 80, 75, 65, 60 and 55 mmHg. We evaluated the longest continuous episode below each threshold during vasopressor therapy. The primary outcome was ICU mortality.

**Results:**

Of 5347 patients with distributive shock, 95.7%, 91.0%, 62.0%, 36.0% and 17.2%, respectively, had MAP < 80, < 75, < 65, < 60 and < 55 mmHg for more than two consecutive hours. On average, ICU mortality increased by 1.3, 1.8, 5.1, 7.9 and 14.4 percentage points for each additional 2 h with MAP < 80, < 75, < 65, < 60 and < 55 mmHg, respectively. Multivariable logistic modeling showed that, compared to patients in whom MAP was never < 65 mmHg, ICU mortality increased as duration of hypotension < 65 mmHg increased [for > 0 to < 2 h, odds ratio (OR) 1.76, *p* = 0.005; ≥ 6 to < 8 h, OR 2.90, *p* < 0.0001; ≥ 20 h, OR 7.10, *p* < 0.0001]. When hypotension was defined as MAP < 60 or < 55 mmHg, the associations between duration and mortality were generally stronger than when hypotension was defined as MAP < 65 mmHg. There was no association between hypotension and mortality when hypotension was defined as MAP < 80 mmHg.

**Conclusions:**

Within the limitations due to the nature of the study, most patients with distributive shock experienced at least one episode with MAP < 65 mmHg lasting > 2 h. Episodes of prolonged hypotension were associated with higher mortality.

**Electronic supplementary material:**

The online version of this article (10.1186/s13613-018-0448-9) contains supplementary material, which is available to authorized users.

## Introduction

Shock is a state of acute circulatory failure characterized by inadequate tissue oxygen delivery, resulting in end-organ dysfunction and high risk of death [1–3]. Distributive shock, characterized by a fall in vascular tone, is the most common form, accounting for approximately two-thirds of all cases of shock [4, 5].

For patients in septic shock, even relatively brief periods of hypoperfusion are associated with poor outcomes [6]. When mean arterial pressure (MAP) is below a certain threshold, organ blood flow falls linearly. The Surviving Sepsis Campaign (SSC) guidelines [5] call for an initial MAP target of 65 mmHg for patients with septic shock, followed by individual titration of vasopressor agents. Although there is a general consensus that MAP should be maintained > 65 mmHg [5–8], there is debate regarding need for higher MAP targets in some patients [9–14].

The objectives of this study were twofold. First, we sought to describe the frequency and duration of hypotensive episodes in patients with distributive shock at a highly reputed academic medical center. Second, we aimed to understand the association between prolonged episodes of hypotension and mortality. We hypothesized that the severity and duration of hypotension would be independently associated with mortality risk in patients with distributive shock.

## Materials and methods

### Data source

Data were obtained from the Medical Information Mart for Intensive Care (MIMIC-III, Version 1.4), which contains comprehensive, time-stamped information for > 60,000 ICU admissions at Beth Israel Deaconess Medical Center (BIDMC) in Boston, Massachusetts between 2001 and 2012, representing > 46,000 unique patients [15]. MIMIC-III data are Health Insurance Portability and Accountability Act of 1996 (HIPAA) compliant, and all investigators with data access (MEG, RD) were approved by PhysioNet.

Information available in MIMIC-III includes dates of admission to the intensive care unit (ICU) and hospital, sex, and dates of birth, transfer and discharge. Clinical elements include charted clinical observations, laboratory and microbiology test results, prescriptions, fluid balance, physiological scores, primary and secondary diagnosis codes (in International Classification of Diseases 9th Edition, Clinical Modification [ICD-9-CM] format), diagnosis-related groups (DRG), procedure codes (in Current Procedural Terminology [CPT] format), and mortality (in-hospital as well as post-discharge).

Yearly data were not available because of data privacy concerns. We only knew whether an admission occurred before or after mid-2008 when new data management software was installed, but the specific date of installation was not available.

### Study subjects

Patients for this study were selected from all persons in MIMIC-III aged ≥ 18 years at ICU admission who had received vasopressors (norepinephrine, epinephrine, dopamine, phenylephrine and/or vasopressin) continuously for ≥ 6 h between ICU admission and discharge, defined as the earliest of recorded ICU discharge, hospital discharge or time of death.

Of these patients, we excluded those with conditions that may be associated with nondistributive shock, including: (1) cardiogenic shock (ICD-9-CM diagnosis codes 785.51 or 998.01), cardiac tamponade (423.3), pulmonary embolus (415.1); (2) use of intra-aortic balloon pump (IABP) or extracorporeal membrane oxygenation (ECMO); (3) administration of ≥ 3500 mL of red blood cells during any 48-h period; or (4) evidence (based on DRG) of specific procedures (e.g., cardiovascular, extra-cranial vascular). We also excluded patients with gaps in their ICU stay data as well as those with more than one otherwise qualifying ICU stay in a given hospital admission.

### Measures

We used norepinephrine-equivalent dose (NED) to calculate the vasopressor dose [16]. We identified a priori a “high-dose” subgroup that received vasopressors at NED ≥ 0.2 μg/kg/min over any 6-h period between ICU admission and discharge [17].

MAP was recorded roughly every hour from invasive arterial line recordings. Hypotension time was defined as a patient’s longest continuous episode with MAP < 65 mmHg over the entire duration of vasopressor therapy (hypotensive episodes occurring in the absence of vasopressor support were not considered). The pre-defined MAP value of 65 mmHg was chosen in order to be consistent with current recommendations [5–8]. To test the sensitivity of our findings with respect to a threshold of 65 mmHg, additional analyses were conducted using alternative thresholds of 55 mmHg, 60 mmHg, 75 mmHg and 80 mmHg.

Previous research on hypotension and mortality used average MAP during the ICU stay to measure hypotension [1, 2, 18]. We reasoned that a patient’s longest continuous episode might better capture the potential damage resulting from hypotension because staying below threshold prevents recovery and organ perfusion. In addition, measurement of the longest continuous episode of hypotension is easier to understand and to monitor in a clinical setting than more complicated measures, such as time-weighted average MAP [13].

Mortality was assessed at ICU discharge. Patients were designated dead at discharge from the ICU if an in-hospital date of death was noted within 24 h of ICU discharge. Otherwise, patients were considered to be alive.

Severity of illness was captured by Sequential Organ Failure Assessment (SOFA) scores, use of mechanical ventilation, use of renal replacement therapy (RRT), baseline lactate, albumin and creatinine concentrations, and the highest dose of catecholamines recorded during the ICU stay [19, 20].

### Statistical analyses

MAP, blood lactate, albumin concentration, creatinine, use of mechanical ventilation, use of RRT and SOFA score were identified from MIMIC-III. DRGs and principal diagnosis codes (ICD-9-CM) were also recorded.

To evaluate the prevalence and severity of hypotension in the ICU, we provide descriptive statistics on the percentage of patients with MAP < 80 mmHg, < 75 mmHg, < 65 mmHg, < 60 mmHg and < 55 mmHg. Because brief transient episodes of hypotension occur frequently in clinical practice and are unlikely to have the same prognostic significance as more sustained episodes, we were primarily interested in episodes of hypotension that lasted at least 2 h. In addition, we stratified occurrence of hypotension by two periods, before and after mid-2008, to explore whether there was any temporal change in the occurrence of hypotension.

ICU mortality was assessed in relation to the longest episode of hypotension during the ICU stay following initiation of vasopressor therapy. Ninety-five percent confidence intervals were calculated using the Wilson score interval [21]. Multivariable logistic regression was used to study the relationship between the longest continuous episode with MAP below threshold and ICU mortality. In this regression, the outcome variable was ICU mortality, and the key explanatory variable was the longest episode of continuous hypotension < 65 mmHg. Major control variables included baseline MAP, age, sex, sepsis, highest catecholamine dose, baseline mechanical ventilation status, baseline RRT status, SOFA score, and baseline lactate, albumin and creatinine concentrations.

Data are presented as mean ± SD. All data analyses were conducted using SAS software, version 9.4.

## Results

### Baseline characteristics

Of the 61,532 ICU admissions included in the MIMIC-III database, 5347 met all the criteria for inclusion (Additional file 1: Figure S1); 2066 of these admissions were designated “high-dose.” Mean patient age was 66 years and mean SOFA score 7.4 ± 3.8 (Table 1). At the start of vasopressor therapy, 30.8%, 19.4% and 11.3% of patients had MAP < 65, < 60 and < 55 mmHg, respectively. Corresponding percentages for the high-dose vasopressor subgroup were 35.7%, 23.6% and 13.8%. In 6.2% of patients, there were no data on MAP values prior to initiation of vasopressor therapy. The top five DRGs and top five principal diagnoses are shown in Additional file 1: Table S1.

**Table 1:** Demographic and clinical characteristics of intensive care unit patients with distributive shock in the MIMIC-III database, 2001–2012

Characteristic	High-dose subgroup^a^	Non-high dose	All patients
	(*N* = 2066)	(*N* = 3281)	(*N* = 5347)
Sex *N* (%)			
Female	943 (45.6)	1548 (47.2)	2491 (46.6)
Male	1123 (54.4)	1733 (52.8)	2856 (53.4)
Age, years			
Mean (SD)	65.4 (15.8)	66.8 (15.6)	66.3 (15.7)
Body weight (kgs)^b^			
Mean (SD)	81.3 (25.3)	81.8 (25.2)	81.6 (25.2)
Missing, *N* (%)	26 (1.3)	31 (0.9)	57 (1.1)
Mean arterial pressure (MAP)^b^			
Mean (SD)	72.5 (18.9)	75.9 (20.1)	74.6 (19.7)
Missing, *N* (%)	120 (5.8)	209 (6.4)	329 (6.2)
Number (%) of patients with			
< 65 mmHg	694 (35.7)	852 (27.7)	1546 (30.8)
< 60 mmHg	459 (23.6)	515 (16.8)	974 (19.4)
< 55 mmHg	269 (13.8)	296 (9.6)	565 (11.3)
Lactate concentration (mmol/L)^b^			
Mean (SD)	3.7 (3.0)	2.5 (2.1)	3.0 (2.6)
Missing, *N* (%)	501 (24.2)	866 (26.4)	1367 (25.6)
Albumin concentration (g/L)^b^			
Mean (SD)	2.7 (0.7)	2.9 (0.7)	2.9 (0.7)
Missing, *N* (%)	1103 (53.4)	2019 (61.5)	3122 (58.4)
Creatinine (mg/dL)^b^			
Mean (SD)	2.1(1.9)	1.9 (1.8)	2.0 (1.9)
Missing, *N* (%)	114 (5.5)	180 (5.5)	294 (5.5)
Baseline mechanical ventilation status^b^			
No, *N* (%)	880 (42.6)	1725 (52.6)	2605 (48.7)
Yes, *N* (%)	1186 (57.4)	1556 (47.4)	2742 (51.3)
Baseline continuous renal replacement therapy^b^			
No, *N* (%)	2046 (99.0)	3261 (99.4)	5307 (99.3)
Yes, *N* (%)	20 (1.0)	20 (0.6)	40 (0.7)
SOFA score^b^			
Mean (SD)	9.0 (4.0)	6.4 (3.2)	7.4 (3.8)
Hypertension			
No, *N* (%)	1457 (70.5)	2159 (65.8)	3616 (67.6)
Yes, *N* (%)	609 (29.5)	1122 (34.2)	1731 (32.4)
Severe sepsis or septic shock			
No, *N* (%)	1195 (36.4)	389 (18.8)	1584 (29.6)
Yes, *N* (%)	2086 (63.6)	1677 (81.2)	3763 (70.4)
Maximum NED during treatment			
Mean (SD)	1.18 (3.45)	0.32 (2.44)	0.65 (2.90)

*NED* norepinephrine-equivalent dose

^a^High-dose vasopressor subgroup is defined as NED ≥ 0.2 µg/kg/min for ≥ 6 h between ICU admission and discharge

^b^At initiation of vasopressor therapy

The average time between MAP readings was 47.5 ± 12.0 min; in 95% of patients, it was < 61.3 min and in 99% < 71.1 min.

### Occurrence of hypotension

Overall, 93.4% of patients had at least one documented MAP reading < 65 mmHg: 95.7%, 91.0%, 62.0%, 36.0% and 17.2% had an episode of MAP continuously < 80 mmHg, < 75 mmHg, < 65 mmHg, < 60 mmHg and < 55 mmHg, respectively, for at least 2 consecutive hours (Table 2). There was little difference in the frequency or severity of hypotensive episodes before versus after mid-2008 (Table 2).

**Table 2:** Percentage of patients with longest continuous episode of mean arterial pressure (MAP) below 80 mmHg, 75 mmHg, 65 mmHg, 60 mmHg, and 55 mmHg over total intensive care unit stay and dosing of vasopressors in patients with distributive shock in the MIMIC-III database, for the periods of 2001–2012, 2001–2008, and 2008–2012

Measure	Ever below	≥ 2 h	≥ 4 h	≥ 8 h	≥ 12 h	≥ 16 h	≥ 20 h
Continuous time below MAP threshold during vasopressor therapy 2001–2012							
All patients, % (*N* = 5347)							
MAP < 80 mmHg	99.3	95.7	87.3	67.0	49.8	37.3	28.6
MAP < 75 mmHg	98.7	91.0	77.4	51.1	34.1	23.4	16.7
MAP < 65 mmHg	93.4	62.0	35.0	14.1	7.3	4.1	2.7
MAP < 60 mmHg	83.7	36.0	15.2	5.3	2.6	1.5	0.9
MAP < 55 mmHg	67.6	17.2	6.0	2.4	1.2	0.6	0.4
Continuous time below MAP threshold during vasopressor therapy 2001–2008							
All patients, % (*N* = 3173)							
MAP < 80 mmHg	99.1	95.1	86.4	67.0	50.4	38.3	29.6
MAP < 75 mmHg	98.1	89.9	76.3	51.3	34.8	24.3	17.6
MAP < 65 mmHg	92.1	60.7	35.0	15.0	8.0	4.6	2.9
MAP < 60 mmHg	81.1	35.7	15.6	6.0	3.1	1.7	0.9
MAP < 55 mmHg	64.5	17.8	6.6	2.8	1.3	0.7	0.4
Continuous time below MAP threshold during vasopressor therapy 2008–2012							
All patients, % (*N* = 2174)							
MAP < 80 mmHg	99.8	96.7	88.6	67.1	48.9	35.9	27.0
MAP < 75 mmHg	99.4	92.5	79.0	50.8	32.9	22.2	15.3
MAP < 65 mmHg	95.3	63.8	35.1	12.7	6.3	3.4	2.3
MAP < 60 mmHg	87.4	36.7	14.6	4.3	1.9	1.2	0.9
MAP < 55 mmHg	72.1	16.4	5.3	1.9	1.1	0.5	0.4

### Relationship between hypotension and mortality

Overall ICU mortality was 29.4%. In patients whose longest episode of hypotension was < 2 h with MAP < 65 mmHg, < 60 mmHg or < 55 mmHg, mortality rates were 20.0%, 22.7% and 24.9%, respectively. In contrast, the corresponding mortality rates for patients whose longest episode of hypotension was ≥ 2 h were 35.1%, 41.3% and 51.0% (*p* < 0.0001 for all comparisons of < 2 h vs. ≥ 2 h below each threshold).

**Fig. 1:**
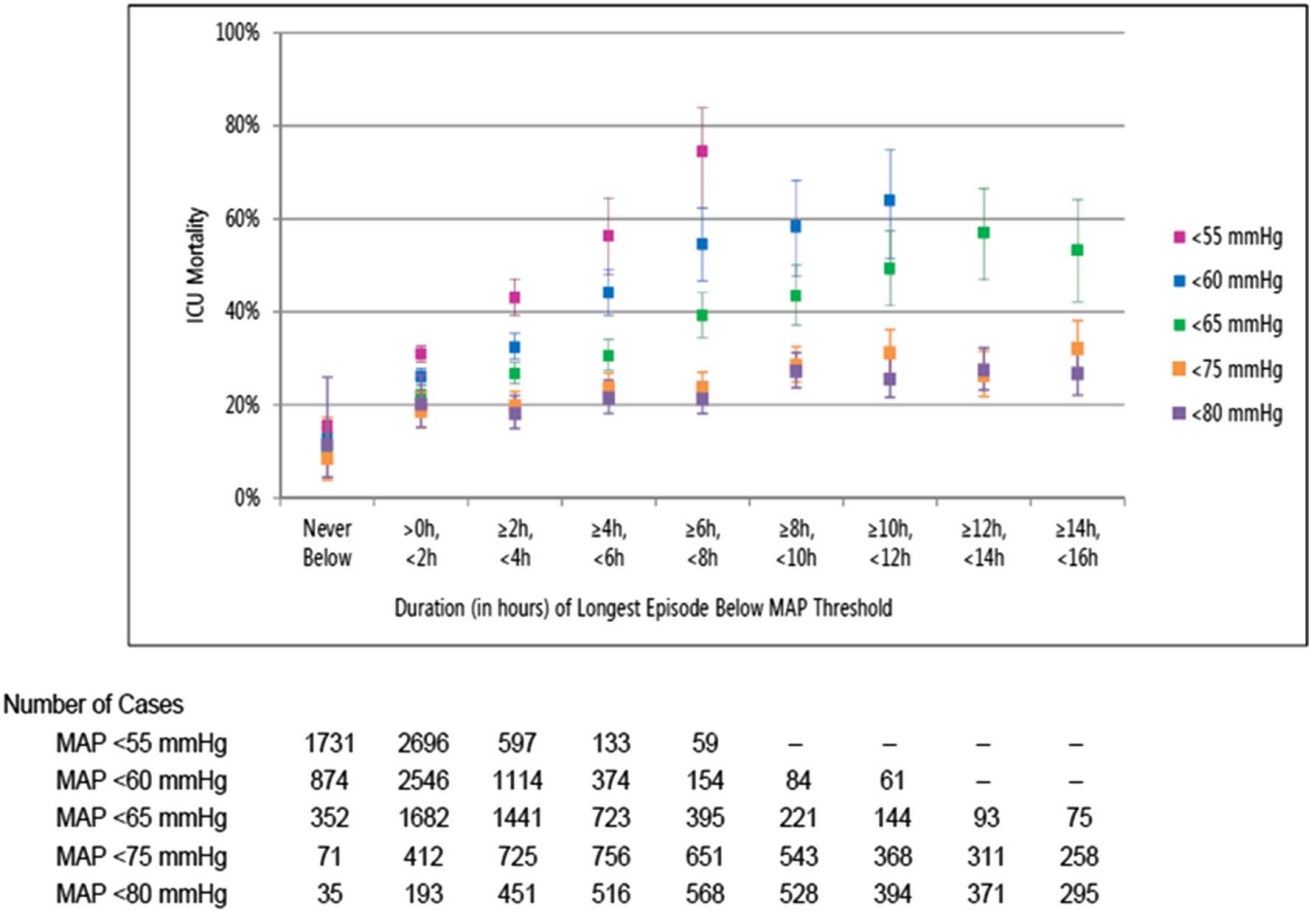
ICU mortality by duration of longest episode with mean arterial pressure (MAP) < 80 mmHg (mauve), < 75 mmHg (orange), < 65 mmHg (green), 60 mmHg (blue) and 55 mmHg (pink) in all patients with distributive shock

ICU mortality rates increased with duration of hypotensive episodes (Fig. 1). For patients whose MAP was never < 80, < 75, < 65, < 60 or < 55 mmHg, ICU mortality rates were 11.4%, 8.5%, 10.2%, 12.6% and 15.4%, respectively. In patients whose longest episode of hypotension was > 0 h but < 2 h, mortality rates increased to 20.2%, 18.7%, 22.1%, 26.1% and 31.0% for MAP < 80, < 75, < 65, < 60 and < 55 mmHg, respectively. In patients with a ≥ 6 h to < 8 h episode of hypotension, mortality rates were 21.3%, 23.8%, 39.2%, 54.5% and 74.6% for the thresholds of 80, 75, 65, 60 and 55 mmHg, respectively. The trends for patients with one or more episodes of hypotension indicate that ICU mortality rates increased by 1.3, 1.8, 5.1, 7.9 and 14.4 percentage points for each additional 2 h with MAP < 80, < 75, < 65, < 60 and < 55 mmHg, respectively. Similar patterns were observed in patients in the high-dose vasopressor subgroup (Additional file 1: Figure S2).

Because it is possible that an early hypotensive episode could change a patient’s illness trajectory, as a sensitivity analysis, we assessed ICU mortality in a cohort of patients excluding the 198 (3.7%) patients who died within the first 24 h after ICU admission. This did not meaningfully change the results (Additional file 1: Figure S3).

In multivariable logistic regression, hypotension time < 65 mmHg was strongly predictive of ICU mortality (Table 3). The ORs associated with MAP < 65 mmHg increased steadily as the hypotension time increased: 1.76 (*p* = 0.005) for continuous hypotension time > 0 and < 2 h, 2.90 (*p* < 0.0001) for continuous hypotension time ≥ 6 and < 8 h, 5.30 (*p* < 0.0001) for continuous hypotension time ≥ 12 and < 16 h and 7.10 (*p* < 0.0001) for ≥ 20 h. The predicted ICU mortality increased from 13.9% for patients who never had MAP < 65 mmHg to 31.9% for patients with hypotension time ≥ 6 and < 8 h, and 53.4% for patients with hypotension time ≥ 20 h.

**Table 3:** Multivariable logistic regression analyzing intensive care unit mortality and the longest episode of hypotension time below threshold of 65 mmHg

Parameter	Estimate	Standard error	Wald Chi-square	Pr > ChiSq	Odds ratio
Intercept	− 3.39	0.23	219.19	< 0.0001	0.03
MAP at initiation of vasopressors (vs. ≥ 65 mmHg)					
< 65 mmHg	0.15	0.07	4.29	0.0384	1.16
Missing	− 0.36	0.16	5.10	0.024	0.70
Longest episode with MAP < 65 mmHg (vs. never below), hours					
> 0 to < 2	0.57	0.20	7.88	0.005	1.76
≥ 2 to < 4	0.66	0.20	10.38	0.0013	1.93
≥ 4 to < 6	0.80	0.21	14.19	0.0002	2.23
≥ 6 to < 8	1.06	0.23	22.15	< 0.0001	2.90
≥ 8 to < 10	1.24	0.25	25.04	< 0.0001	3.44
≥ 10 to < 12	1.47	0.27	29.19	< 0.0001	4.36
≥ 12 to < 16	1.67	0.26	40.46	< 0.0001	5.30
≥ 16 to < 20	1.70	0.32	27.87	< 0.0001	5.47
≥ 20	1.96	0.28	49.20	< 0.0001	7.10
Max catecholamine dose (vs. 0 to < 0.2), NED					
≥ 0.2 to < 0.5	0.64	0.09	50.58	< 0.0001	1.89
≥ 0.5 to < 1.0	1.44	0.10	195.55	< 0.0001	4.24
≥ 1.0 to < 1.5	1.85	0.14	174.34	< 0.0001	6.37
≥ 1.5 to < 2.0	2.07	0.24	73.03	< 0.0001	7.95
≥ 2.0 to < 2.5	2.08	0.31	45.37	< 0.0001	7.97
≥ 2.5	1.61	0.20	63.21	< 0.0001	4.99
Age (vs. < 65), years					
65–74	0.33	0.09	12.48	0.0004	1.39
75–84	0.48	0.09	28.23	< 0.0001	1.62
≥ 85	0.73	0.11	44.40	< 0.0001	2.07
Female (vs. male)	− 0.07	0.07	0.92	0.3364	0.94
Severe sepsis/septic shock (vs. none)	− 0.19	0.08	5.44	0.0197	0.83
SOFA score (vs. ≤ 4)					
> 4 to ≤ 6	0.03	0.11	0.07	0.7851	1.03
> 6 to ≤ 8	0.09	0.11	0.58	0.4447	1.09
> 8 to ≤ 10	0.32	0.12	6.75	0.0094	1.37
> 10	1.09	0.12	83.90	< 0.0001	2.98
Hypertension (vs. none)	− 0.02	0.08	0.09	0.7674	0.98
Baseline mechanical ventilation (vs. none)	0.15	0.07	4.45	0.035	1.16
Baseline continuous renal replacement therapy (vs. none)	1.26	0.38	11.19	0.0008	3.54
Albumin level (vs. ≥ 2.5 g/dL)					
< 2.5 g/dL	0.60	0.11	29.41	< 0.0001	1.83
Missing	0.18	0.08	5.01	0.0252	1.20
Creatinine level (vs. < 2.0 mg/dL)					
≥ 2.0 mg/dL	− 0.09	0.08	1.14	0.2863	0.92
Missing	− 0.55	0.17	10.07	0.0015	0.57
Lactate level (vs. < 2.0 mmol/L)					
≥ 2.0 mmol/L	0.38	0.08	20.66	< 0.0001	1.46
Missing	0.37	0.10	14.90	0.0001	1.45

The highest catecholamine dose was also strongly significant in predicting ICU mortality (Table 3). The predicted marginal probabilities of ICU mortality by highest catecholamine dose were 14.7% for patients with a dose of < 0.2 NED and 53.8% for patients with doses of ≥ 1.0 and < 1.5 NED (Additional file 1: Figure S4).

We performed additional modeling analyses using time below MAP of 55, 60, 75 and 80 mmHg as measures of hypotension: in general, when the threshold was < 65 mmHg, the ORs for mortality associated with hypotension time were stronger than with higher thresholds (Table 4). When the threshold was increased to 75 mmHg, the association was partly attenuated; however, ORs trended higher with longer episodes of hypotension, becoming statistically significant at 16 h. At a threshold of 80 mmHg, there was no association between hypotension and mortality.

**Table 4:** Multivariable logistic regression analyzing ICU mortality and the longest episode of hypotension below thresholds of 50 mmHg, 60 mmHg, 75 mmHg, and 80 mmHg

Parameter	MAP < 55 mmHg		MAP < 60 mmHg		MAP < 75 mmHg		MAP < 80 mmHg	
	Pr > ChiSq	Odds Ratio	Pr > ChiSq	Odds Ratio	Pr > ChiSq	Odds Ratio	Pr > ChiSq	Odds Ratio
Longest episode with MAP below threshold (vs. never below), hours								
> 0 to < 2	< 0.0001	1.71	< 0.0001	1.71	0.2556	1.70	0.5474	1.42
≥ 2 to < 4	< 0.0001	2.67	< 0.0001	1.99	0.2858	1.63	0.895	1.08
≥ 4 to < 6	< 0.0001	4.41	< 0.0001	3.50	0.1895	1.82	0.9018	1.07
≥ 6 to < 8	< 0.0001	12.06	< 0.0001	4.80	0.2362	1.72	0.8184	1.14
≥ 8 to < 10	< 0.0001	7.18	< 0.0001	5.65	0.0795	2.23	0.5034	1.46
≥ 10 to < 12	0.0008	5.68	< 0.0001	6.58	0.0788	2.25	0.7379	1.21
≥ 12 to < 16	< 0.0001	8.17	< 0.0001	7.50	0.1023	2.11	0.6686	1.27
≥ 16 to < 20	0.0084	7.30	< 0.0001	7.37	0.0135	3.13	0.5393	1.41
≥ 20	0.0004	6.73	< 0.0001	7.32	0.0035	3.75	0.1943	2.06

We also performed sensitivity analyses by (1) excluding patients before 2008; (2) using 28-day mortality as the outcome variable; and (3) defining hypotension intervals in episodes of 6 h instead of 2 h (0–6 h, 6–12 h, 12–24 and > 24 h). These analyses did not alter our findings (data not shown).

## Discussion

Our study brings further evidence that prolonged episodes of hypotension are common in patients with distributive shock; in this large database, 62.0% of patients had MAP continuously < 65 mmHg and 17.2% < 55 mmHg for at least 2 h during their ICU stay. In addition, the frequency of hypotensive episodes did not change after introduction of the first SSC guideline in 2004 [22], which stressed the importance of maintaining a MAP of at least 65 mmHg, with no difference in frequency before and after mid-2008. Unlike clinical trials, which have stringent inclusion and exclusion criteria, our dataset included almost all patients with distributive shock in a real-world setting.

There are several possible reasons for these common episodes of hypotension including concern about severe adverse events, such as cardiac injury, mesenteric and digital ischemia, particularly when vasopressor doses are high [19, 20, 23, 24]. Whatever the reason, our results support the association of hypotension with poor clinical outcomes. The amount of time spent continuously below a MAP threshold of 65 mmHg was strongly predictive of mortality. Each additional 2-h increment in the longest episode under threshold was associated with a progressive increase in mortality rate. This observed relationship between hypotension and mortality suggests that the development of any hypotensive episodes while on vasopressor support should be closely monitored and aggressive treatment may be warranted to correct these episodes.

Prior studies evaluating this relationship have generally been limited by small sample sizes and yielded conflicting results [1, 2]. One recent study used a larger dataset to investigate similar questions [13], but did not adjust for some important mortality predictors, including catecholamine doses [19, 20, 23], and use of mechanical ventilation or RRT [25, 26]. By accounting for such variables, we therefore provide a more robust analysis of the relationship between hypotension and patient outcomes. Indeed, the observed association between hypotension and ICU mortality is generally stronger than that reported in these previous studies [1, 13]. In the study by Dunser et al. [1], an episode of MAP < 60 mmHg in the first 24 h was associated with higher mortality than no such episode; in our study, any episode of MAP < 65 mmHg was associated with higher mortality. In the recent study by Maheshwari et al. [13], ORs for ICU mortality increased by 1.037 for every 2-h increase in cumulative time < 65 mmHg. In our study, ORs for ICU mortality increased by 1.092–1.313 for every 2-h increase in continuous duration < 65 mmHg. Importantly, the magnitude of the OR increase remained even when we removed variables not included in the study by Maheshwari et al. [13], such as catecholamine dose, baseline mechanical ventilation and baseline RRT status. Maheshwari et al. [13] also reported a significant association between hypotension and mortality even when the MAP threshold was raised to 85 mmHg, whereas we found no such relationship when the hypotension threshold was 80 mmHg. The difference in cumulative time vs continuous time may in part explain these differences.

The findings in this study provide some evidence for the initial target of MAP 65 mmHg given in the SSC guidelines [5]. There has been much debate on the appropriate MAP target for patients with distributive shock. The seminal study by Asfar et al. showed that there was no mortality difference between a lower (65–70 mmHg) and a higher (80–85 mmHg) MAP target [9]. In our study, at a threshold of < 75 mmHg, ORs for mortality trended to higher values with longer episodes of hypotension and were statistically significant at 16 h. At 80 mmHg, there was no association between hypotension and mortality. When the hypotension thresholds were reduced to 60 mmHg or 55 mmHg, the association between hypotension and mortality was further strengthened. Our data therefore suggest that, for populations of critically ill patients, a target of 65–70 mmHg appears to be a critical threshold at which outcomes are affected, and a higher target of 75 mmHg may even be advisable. A more nuanced view would be that management should be personalized with certain patients requiring a higher target MAP and all patients appearing to be at risk at MAPs that are < 55–60 mmHg.

Our study shows that higher catecholamine dose has a negative impact on mortality risk even when adjusted for duration of hypotension and other covariates. This increased hazard with high-dose catecholamines is plausible given that catecholamines have a narrow therapeutic window. High-dose catecholamines are associated with excessive catalytic free iron, immunosuppression, and microcirculatory defects [27]. Because episodes of hypotension are often positively associated with catecholamine dose, it is important to consider both variables to see whether the hypotension and high-dose catecholamine effects overlap. Most previous studies on ICU mortality for patients with distributive shock considered just the impact of hypotension [1, 9] or the impact of high-dose catecholamines [20, 23]. The study by Varpula et al. examined both mean MAP and peak catecholamine dose as predictors of 30-day mortality [2], but peak catecholamine dose was not a significant predictor. These studies left open the question of whether the observed high-dose catecholamine effect may overlap with the observed effect of hypotension. We showed that hypotensive episodes and catecholamine dose are both strong independent predictors of ICU mortality. This finding suggests that clinicians need to consider the risks of both hypotension and high-dose catecholamines when treating patients with distributive shock.

Our results have important implications for the design of randomized controlled trials in shock. Previous trials on shock, such as Vasopressin versus Norepinephrine as Initial Therapy in Septic Shock (VANISH) and the Vasopressin and Septic Shock Trial (VASST), reported no differences in mortality between treatment groups (vasopressin vs. norepinephrine) [28, 29]. However, these trials did not report the proportions of patients with episodes of hypotension and MAP may not have been uniformly maintained in all patients. Our findings suggest that for future trials in distributive shock, hypotension data should be documented and analyzed in each treatment group as a potential confounder to the primary outcome because we showed that lack of maintenance of MAP was associated with increased mortality.

Our study has several limitations. First, the sensitivity and specificity of our criteria for identifying patients with distributive shock are unknown; our cohort may have included some patients with other forms of shock and excluded some with distributive shock. Second, we used data from a single academic medical center in the USA, with the earliest cases from almost 20 years ago, when care may have been inconsistent with currently accepted standards. The single-center nature of the study may also limit the applicability of our findings to other sites. Nevertheless, a single-center study increases the likelihood of more uniform patient treatment, reducing concerns that observed mortality differences may be caused by practice differences among centers. Third, because of the retrospective nature, some important data elements were not available. For example, it was unclear whether prolonged episodes of hypotension were caused by lack of treatment or some other reason, such as permissive hypotension, because MAP target goals were not recorded. Intravascular volume status, global oxygen delivery, perfusion pressure and central venous pressure were other potentially important data elements that could not be included in the study because they were not consistently recorded. Furthermore, patients were not randomized into different hypotension groups, so the observed relationship between hypotension and mortality indicates association, not causation. In addition, because patients may be present in multiple hypotension groups (similar to the research design of the study by Maheshwari et al. [13] in which the patients within the 75 mmHg group consisted of those in the 65 mmHg group in addition to those in the 75 mmHg group for the relevant duration), it was difficult to identify the marginal effect on mortality of each successive threshold. Fourth, some of our control variables, such as SOFA scores, mechanical ventilation status, RRT status and levels of lactate, albumin and creatinine were baseline values, although these values may vary considerably during the course of treatment. Finally, our analysis of the relationship between time below MAP thresholds and mortality may be partially biased to the null as a result of the effect of “immortal time bias.” In particular, patients could only have an episode with MAP below threshold for more than a specific time period if they survived sufficiently long to accrue this much time below the threshold. Those who died before accruing this much time below the threshold would have been assigned to groups with a shorter time below threshold. However, the fact that we observed a strong and consistent relationship between time below MAP threshold and ICU mortality, despite the potential effect of immortal time bias, highlights the clinical importance of our observations. Failure to achieve MAP goals over a number of hours in some cases may reflect a deteriorating patient with decreasing vasopressor doses as the transition is gradually made from active therapy to withdrawal and palliative care.

## Conclusions

In this retrospective study in a leading academic center, more than 60% of patients with distributive shock had a MAP < 65 mmHg, and 17.2% had a MAP < 55 mmHg, for at least 2 h during their ICU stay. ICU mortality increased with duration of longest hypotensive episode below threshold, highlighting the potential prognostic importance of hypotensive periods in patients with distributive shock.

## Additional file

**Additional file 1.** Table S1 and Figures S1–4.

## Abbreviations

CPT: current procedural terminology
DRG: diagnosis-related groups
ECMO: extracorporeal membrane oxygenation
IABP: intra-aortic balloon pump
ICD: International Classification of Diseases
ICU: intensive care unit
MAP: mean arterial pressure
MIMIC: Medical Information Mart for Intensive Care
NED: norepinephrine-equivalent dose
OR: odds ratio
RRT: renal replacement therapy
SOFA: sequential organ failure assessment
SSC: Surviving Sepsis Campaign

## References

[1] Dunser MW, Takala J, Ulmer H, Mayr VD, Luckner G, Jochberger S (2009). Arterial blood pressure during early sepsis and outcome. Intensive Care Med.

[2] Varpula M, Tallgren M, Saukkonen K, Voipio-Pulkki LM, Pettila V (2005). Hemodynamic variables related to outcome in septic shock. Intensive Care Med.

[3] Vincent JL, De Backer D (2013). Circulatory shock. N Engl J Med.

[4] De Backer D, Biston P, Devriendt J, Madl C, Chochrad D, Aldecoa C (2010). Comparison of dopamine and norepinephrine in the treatment of shock. N Engl J Med.

[5] Rhodes A, Evans LE, Alhazzani W, Levy MM, Antonelli M, Ferrer R (2017). Surviving Sepsis Campaign: international guidelines for management of sepsis and septic shock: 2016. Crit Care Med.

[6] Waechter J, Kumar A, Lapinsky SE, Marshall J, Dodek P, Arabi Y (2014). Interaction between fluids and vasoactive agents on mortality in septic shock: a multicenter, observational study. Crit Care Med.

[7] Leone M, Asfar P, Radermacher P, Vincent JL, Martin C (2015). Optimizing mean arterial pressure in septic shock: a critical reappraisal of the literature. Crit Care.

[8] Cecconi M, De Backer D, Antonelli M, Beale R, Bakker J, Hofer C (2014). Consensus on circulatory shock and hemodynamic monitoring. Task force of the European Society of Intensive Care Medicine. Intensive Care Med.

[9] Asfar P, Meziani F, Hamel JF, Grelon F, Megarbane B, Anguel N (2014). High versus low blood-pressure target in patients with septic shock. N Engl J Med.

[10] Bourgoin A, Leone M, Delmas A, Garnier F, Albanese J, Martin C (2005). Increasing mean arterial pressure in patients with septic shock: effects on oxygen variables and renal function. Crit Care Med.

[11] Lamontagne F, Meade MO, Hebert PC, Asfar P, Lauzier F, Seely AJE (2016). Higher versus lower blood pressure targets for vasopressor therapy in shock: a multicentre pilot randomized controlled trial. Intensive Care Med.

[12] Thooft A, Favory R, Salgado DR, Taccone FS, Donadello K, De Backer D (2011). Effects of changes in arterial pressure on organ perfusion during septic shock. Crit Care.

[13] Maheshwari K, Nathanson BH, Munson SH, Khangulov V, Stevens M, Badani H (2018). The relationship between ICU hypotension and in-hospital mortality and morbidity in septic patients. Intensive Care Med.

[14] Asfar P, Radermacher P, Ostermann M (2018). MAP of 65: target of the past?. Intensive Care Med.

[15] Johnson AE, Pollard TJ, Shen L, Lehman LW, Feng M, Ghassemi M (2016). MIMIC-III, a freely accessible critical care database. Sci Data.

[16] Chawla LS, Russell JA, Bagshaw SM, Shaw AD, Goldstein SL, Fink MP (2017). Angiotensin II for the treatment of high-output shock 3 (ATHOS-3): protocol for a phase III, double-blind, randomised controlled trial. Crit Care Resusc.

[17] Khanna A, English SW, Wang XS, Ham K, Tumlin J, Szerlip H (2017). Angiotensin II for the treatment of vasodilatory shock. N Engl J Med.

[18] Dunser MW, Ruokonen E, Pettila V, Ulmer H, Torgersen C, Schmittinger CA (2009). Association of arterial blood pressure and vasopressor load with septic shock mortality: a post hoc analysis of a multicenter trial. Crit Care.

[19] Auchet T, Regnier MA, Girerd N, Levy B (2017). Outcome of patients with septic shock and high-dose vasopressor therapy. Ann Intensive Care.

[20] Sviri S, Hashoul J, Stav I, van Heerden PV (2014). Does high-dose vasopressor therapy in medical intensive care patients indicate what we already suspect?. J Crit Care.

[21] Wilson EB (1927). Probable inference, the law of succession and statistical inference. J Am Stat Assoc.

[22] Dellinger RP, Carlet JM, Masur H, Gerlach H, Calandra T, Cohen J (2004). Surviving Sepsis Campaign guidelines for management of severe sepsis and septic shock. Crit Care Med.

[23] Brown SM, Lanspa MJ, Jones JP, Kuttler KG, Li Y, Carlson R (2013). Survival after shock requiring high-dose vasopressor therapy. Chest.

[24] Schmittinger CA, Torgersen C, Luckner G, Schroder DC, Lorenz I, Dunser MW (2012). Adverse cardiac events during catecholamine vasopressor therapy: a prospective observational study. Intensive Care Med.

[25] Leedahl DD, Personett HA, Gajic O, Kashyap R, Schramm GE (2014). Predictors of mortality among bacteremic patients with septic shock receiving appropriate antimicrobial therapy. BMC Anesthesiol.

[26] Mohamed AKS, Mehta AA, James P (2017). Predictors of mortality of severe sepsis among adult patients in the medical intensive care unit. Lung India.

[27] Stolk RF, van der Poll T, Angus DC, van der Hoeven JG, Pickkers P, Kox M (2016). Potentially inadvertent immunomodulation: norepinephrine use in sepsis. Am J Respir Crit Care Med.

[28] Gordon AC, Mason AJ, Thirunavukkarasu N, Perkins GD, Cecconi M, Cepkova M (2016). Effect of early vasopressin versus norepinephrine on kidney failure in patients with septic shock: the VANISH randomized clinical trial. JAMA.

[29] Russell JA, Walley KR, Singer J, Gordon AC, Hebert PC, Cooper DJ (2008). Vasopressin versus norepinephrine infusion in patients with septic shock. N Engl J Med.

