# Supplementary material for: Mean arterial pressure and mortality in patients with distributive shock: a retrospective analysis of the MIMIC-III database
Source: Ann Intensive Care. 2018 Nov 8;8:107. doi: 10.1186/s13613-018-0448-9 (PMC6223403; doi:10.1186/s13613-018-0448-9)
Supplement: Supplementary file 1 — Additional file 1. Table S1 and Figures S1–4. [file 13613_2018_448_MOESM1_ESM.pdf]

Mean Arterial Pressure and Mortality in Patients with Distributive Shock: A Retrospective Study Based on MIMIC-III

Vincent JL et al.

Additional File 1

Fig. S1. Flow chart of patient selection criteria

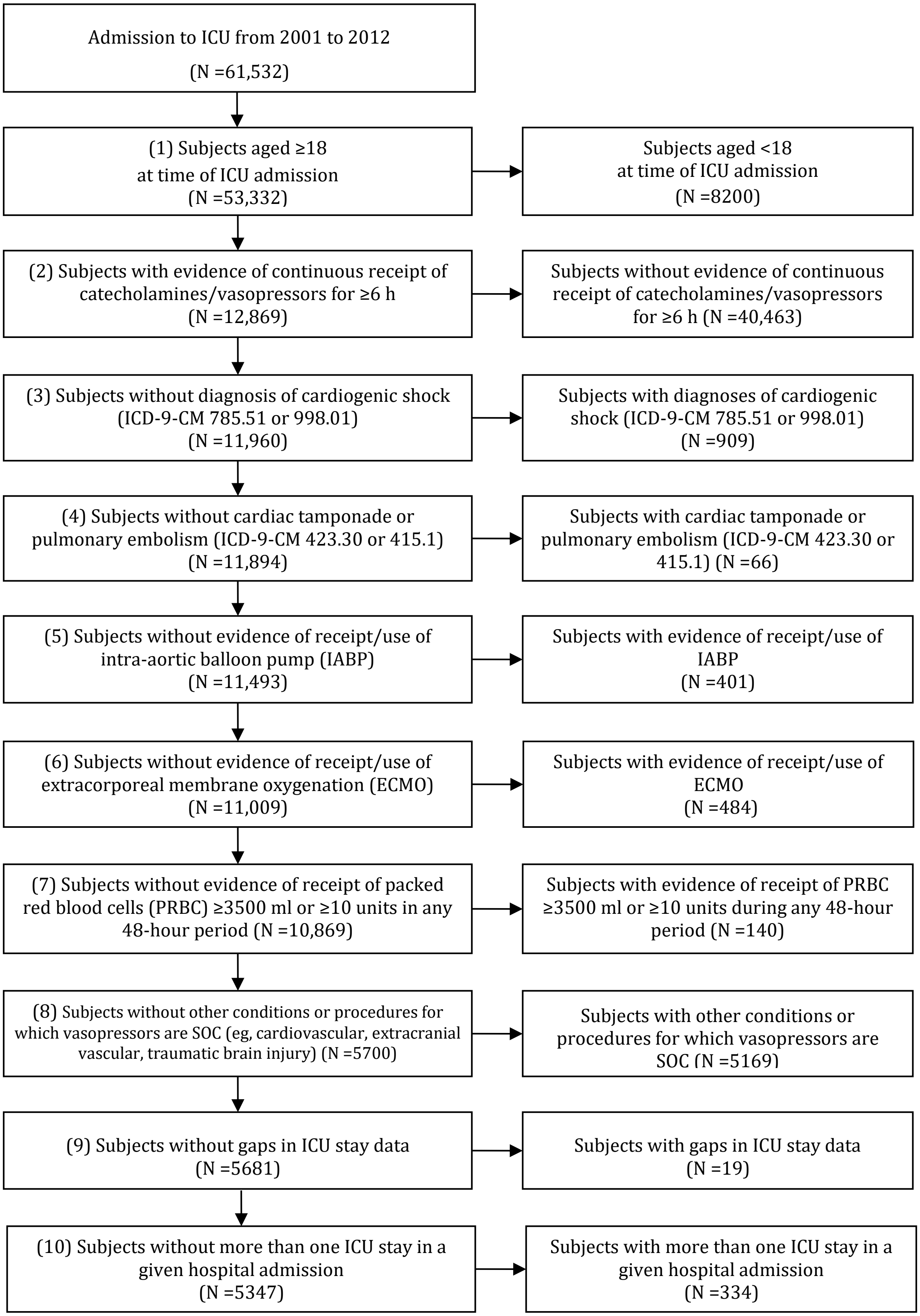

Table S1. Top five diagnosis-related groups and principal diagnoses among all patients and among the high-dose vasopressor subgroup

DRG code	DRG Description	All patients (%)	High-dose subgroup (%)
**Top Five Diagnosis-Related Groups (DRG)**			
DRG 871	Septicemia or severe sepsis without medical ventilation for 96+ hours with MCC	8.8	8.2
DRG 416	Septicemia age >17	7.6	9.8
DRG 475	Respiratory system diagnosis with ventilator support	4.6	4.6
DRG 870	Septicemia or severe sepsis with medical ventilation 96+ hours	3.9	5.7
DRG 853	Infectious and parasitic diseases with operating room procedure with MCC	3.2	3.9
**Top Five Principal Diagnoses**			
ICD-9-CM 038.9	Unspecified septicemia	18.3	22.4
ICD-9-CM 518.81	Acute respiratory failure	4.7	3.9
ICD-9-CM 038.42	Septicemia due to *Escherichia coli*	2.3	2.6
ICD-9-CM 507.0	Pneumonitis due to inhalation of food or vomitus	2.1	-[a]
ICD-9-CM 038.11	Methicillin-susceptible *Staphylococcus aureus* septicemia	1.9	2.6
ICD-9-CM 038.49	Other septicemia due to Gram-negative organisms	-[a]	2.4

[a]Not included as this diagnosis is not in the top five for the group
MCC = major complications/comorbidities

Fig. S2. ICU mortality by duration of longest episode with mean arterial pressure (MAP) <80 mmHg (mauve), <75 mmHg (orange), <65 mmHg (green), 60 mmHg (blue) and 55 mmHg (pink) in all patients in the high-dose vasopressor subgroup

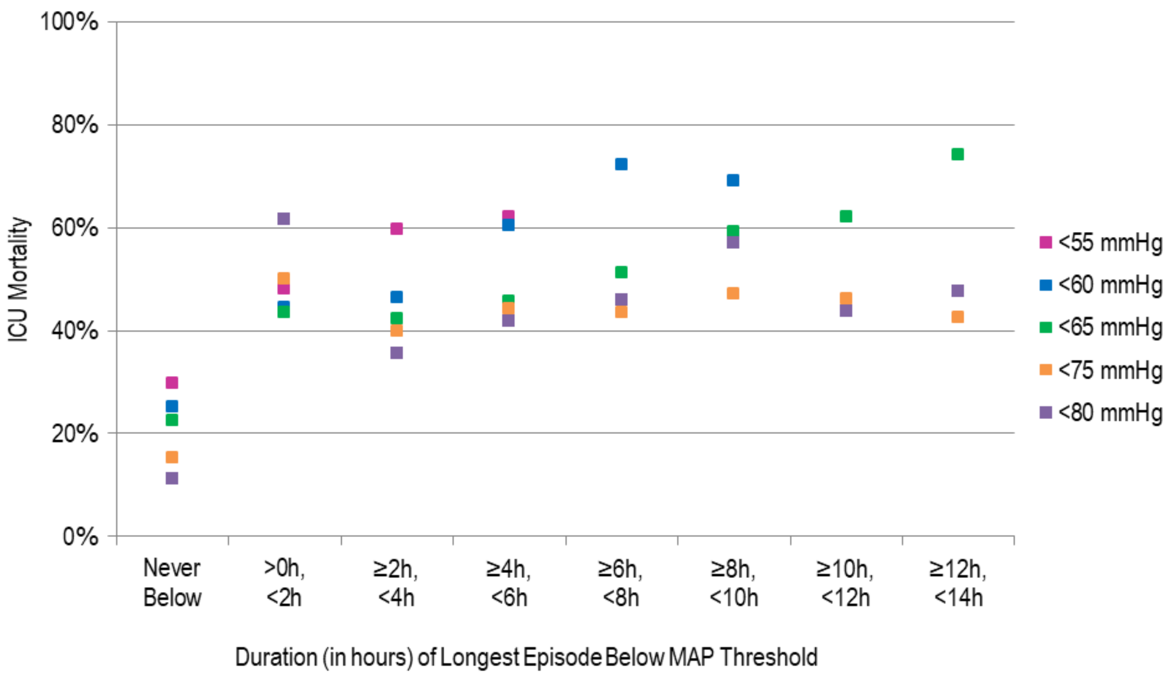

Number of Cases								
MAP <55 mmHg	422	1107	312	87	–	–	–	–
MAP <60 mmHg	171	909	504	195	90	52	–	–
MAP <65 mmHg	58	457	558	321	199	118	87	54
MAP <75 mmHg	13	86	165	222	218	193	145	127
MAP <80 mmHg	9	39	76	124	148	156	142	132

Fig. S3. ICU mortality by duration of longest episode with mean arterial pressure (MAP) <80 mmHg (mauve), <75 mmHg (orange), 65 mmHg (green), 60 mmHg (blue) and 55 mmHg (pink) among patients alive 24 hours after ICU admission

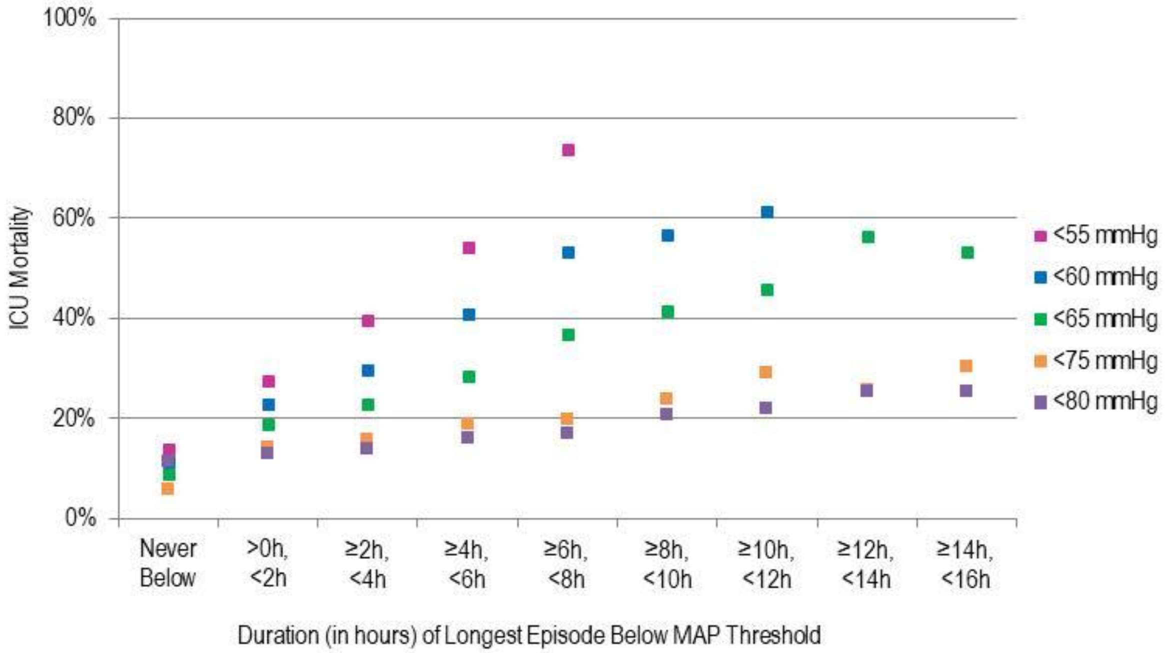

Number of Cases									
MAP <55 mmHg	1702	2572	563	127	57	–	–	–	–
MAP <60 mmHg	863	2438	1069	353	150	81	57	–	–
MAP <65 mmHg	347	1616	1371	701	380	214	135	92	75
MAP <75 mmHg	69	391	689	711	619	510	357	308	251
MAP <80 mmHg	35	177	428	482	538	485	376	360	289

Fig. S4. Marginal probabilities of ICU mortality by maximum catecholamine dose

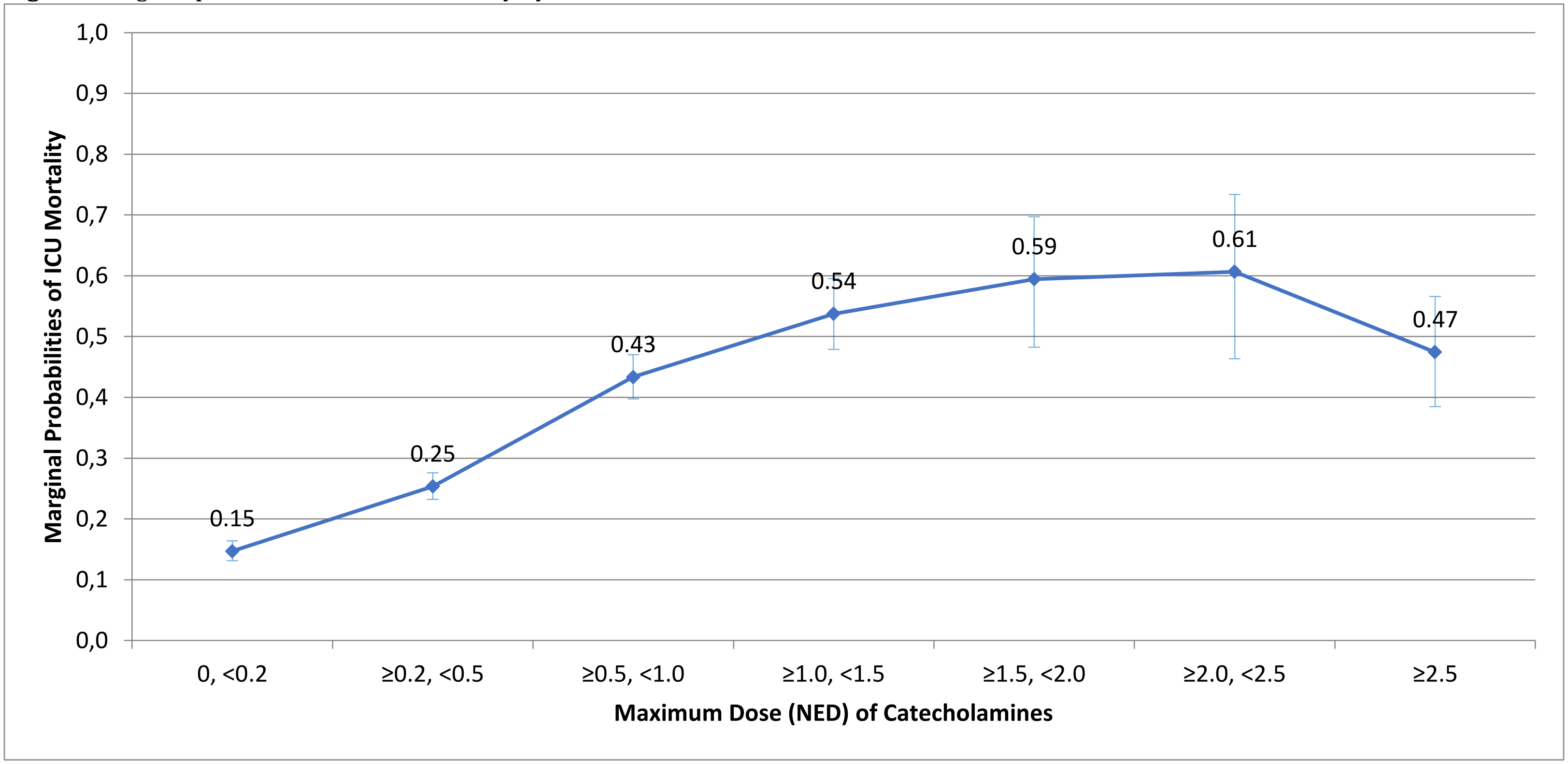

NED: norepinephrine-equivalent dose
